# Psychological Response to COVID-19 in Turkish Dialysis Healthcare Providers

**DOI:** 10.1192/j.eurpsy.2023.1679

**Published:** 2023-07-19

**Authors:** I. Polat, M. S. Sever, E. Demir, H. Yazıcı, S. K. Koç, R. Papila, M. Özkan

**Affiliations:** 1 Istanbul University, Istanbul Faculty of Medicine, Department of Psychiatry; 2 Istanbul University, Istanbul Faculty of Medicine, Department of Internal Medicine, Division of Nephrology; 3 Fresenius Medical Care, Istanbul, Türkiye

## Abstract

**Introduction:**

COVID-19 has been a stressful experience for healthcare providers (HCP), and created an additional distress on dialysis HCP since patients have greater risk of infection, symptom severity and death.

**Objectives:**

We aimed to investigate the level of psychological difficulties in Turkish dialysis HCP during the early outbreak period.

**Methods:**

Participants filled an online survey including a screening questionnaire, Depression Anxiety Stress Scale-21 (DASS-21) and Multidimensional Scale of Perceived Social Support (MSPSS). Chi-Square, Fisher’s exact, Mann- Whitney- U, Kruskal Wallis, Spearman correlation and linear regression analyses were conducted.

**Results:**

Getting infected with COVID-19 and transmitting the disease to their beloveds were the major concerns of HCP. DASS-21 scores were higher in participants who were single and without children, having trouble in finding equipment or worrying about finding equipment in the future, being in contact with COVID-19(+) people; who increased tobacco and alcohol use, and who declared sleep, appetite, somatic problems. Worries about getting COVID-19 [(94.6%) vs. (90.6%) vs. (84.7%); p<0.001] and shortage of equipment [(52.9%) vs. (29.4%) vs. (26.3%); p<0.001]; sleep [(62.2%) vs. (43.5%) vs. (34%); p<0.001] and somatic problems [(58.4%) vs. (50%) vs. (28.2%); p<0.001] and DASS-21 scores [(5-21) vs. (3-15) vs. (0-12); p<0.001] were higher in nurses.

**Conclusions:**

Worries and lifestyle changes associated with the outbreak are related to psychological difficulties. Adequate level of knowledge, self-protection and social support are important issues for HCP. While we recommend the HCP to express and share their worries; institutions should focus on the psychological status of the staff and provide immediate interventions.

**Disclosure of Interest:**

None Declared

